# Fabrication and Properties of Hybrid Coffee-Cellulose Aerogels from Spent Coffee Grounds

**DOI:** 10.3390/polym11121942

**Published:** 2019-11-26

**Authors:** Xiwen Zhang, Li Ping Kwek, Duyen K. Le, Men Shu Tan, Hai Minh Duong

**Affiliations:** 1 Singapore Institute of Manufacturing Technology (SIMTech), Singapore S637662, Singapore; zhangxw@simtech.a-star.edu.sg (X.Z.); Tan_Men_Shu@simtech.a-star.edu.sg (M.S.T.); 2 Department of Mechanical Engineering, National University of Singapore, Singapore S119077, Singapore; kwekliping@u.nus.edu (L.P.K.); mpelkd@nus.edu.sg (D.K.L.); 3 Department for Management of Science and Technology Development, Ton Duc Thang University, Ho Chi Minh City 758307, Vietnam; 4 Faculty of Applied Sciences, Ton Duc Thang University, Ho Chi Minh City 758307, Vietnam

**Keywords:** aerogel, coffee powder, cotton fiber, freeze drying

## Abstract

A fully biodegradable hybrid coffee-cotton aerogel has been successfully developed from spent coffee grounds, 100% cotton fiber and polyvinyl alcohol (PVA) flakes via environmental friendly processes. The cotton fibers in coffee aerogel help to maintain the structure and improve the overall properties of the new hybrid coffee-cotton aerogel. The results show that increasing the concentration of fibers, while keeping the concentration of spent coffee grounds constant, the sinking of coffee ground particles in solution and shrinking effect on the aerogels are minimized and the overall mechanical and oil absorption properties are improved. The developed hybrid aerogels possess high porosity of 92–95% and super-hydrophobicity with an average water contact angle of 139°. Oil absorption capacity achieves 16 g/g with 0.50 wt.% of cotton fibers inside the coffee aerogel. Their thermal conductivity is in the range of 0.037–0.045 W/mK and compressive Young’s modulus achieves highest at 15.6 kPa. The properties of the hybrid aerogel indicate it as a potential material in several applications such as thermal insulation, oil absorption and filtration.

## 1. Introduction

The estimated world coffee consumption in coffee year 2017/18 was 161.93 million bags. This represents an increase of 1.8% as compared with coffee year 2016/17 [1]. With almost 50% of the world coffee production used for soluble coffee preparation, up to 6 million tons of spent coffee grounds (SCG) are generated annually [2]. The presence of natural toxic organic compounds such as caffeine, tannins and polyphenols in SCG make it a pollution hazard when disposed of in the environment [3,4,5]. In addition, the high organic content in SCG requires a huge amount of oxygen to biodegrade it, thus making undesirable its reckless disposal into water [6,7]. Nowadays, the most common way to discard SCG is through landfills. However, this will cause dire effects on the environment due to the enormous amount of methane and carbon dioxide produce when SCG decompose [8].

Recognizing the escalating problem that coffee wastes can cause to the environment, efforts have been undertaken to reuse and recycle SCG. They can be reused as compost and animal feed or recycled for their bioactive compounds and many other applications [8]. However, such efforts are not widely practiced. Thus, to minimize its impact on the environment, it has become the utmost objective to repurpose it into something useful. Due to SCG’s chemical composition, it can exhibit good functional properties such as high water and oil holding capacities [9]. Thus, it has the potential to be incorporated and made into aerogels. Aerogels are materials that exhibit remarkable properties such as extremely low densities and high porosities.

The problems faced with coffee aerogels are the sinking of larger coffee ground particles before the solution is frozen and shrinkage of the resulting coffee aerogels. Therefore, cotton fiber has been introduced to counter this problem. Cotton fiber is made up of 95% cellulose fiber which gives good structural support for the aerogel. Furthermore, when in contact with water, the alignment of polymers will improve, and the number of hydrogen bonds will be higher, which leads to an overall increase of fiber strength. Also, cotton fiber is highly absorbent due to the presence of polar OH groups which attract water molecules [10]. Hence, incorporating cotton fiber into a coffee aerogel to further improve its mechanical properties is an ideal solution. 

Focusing on the problem of oil spills, they are frequent issues in the sea around Singapore given that the Asia Pacific shipping route is one of the busiest sea lanes in the world. It has been reported that the Straits of Singapore is one of the world’s hot spots for oil spills [11]. The current solutions to oil spills are through mechanical treatment, physical, chemical or biological methods. Physical sorbents are the most commonly used because they are abundant, cheap and widely available. There are four types of sorbents: organic sorbents, natural sorbents, inorganic sorbents and synthetic sorbents. Their absorption ratio by weight of oil to sorbent are 10:1, 2:1 and 40:1 respectively [12]. Given that synthetic sorbents are the most effective, it is made up of polyurethane, polyethylene, and polypropylene which are non-biodegradable materials and thus, harmful to the environment [13,14].

Aerogels with a highly interconnected three-dimensional (3D) network, characterized by high porosity, low density, and light weight, have attracted wide interest in the field of oil spill clean-up. Different types of aerogels based on polymers [15], cellulose nanofibrils [16], carbon nanotubes [17], and silica nanoparticles [18] have been recently reported for oil sorption applications in oil/water mixtures. Among these fabricated aerogels, cotton aerogels have been recognized as good oil sorbents as they can absorb a few times more than their weight, given their high porosity and low density [19,20]. In this work, a bio-degradable hybrid coffee-cotton aerogel is developed to alleviate the problem of spent coffee grounds waste and as an alternative for cleaning up oil spills, thus reducing the overall impact caused to the environmental.

## 2. Experimental

### 2.1. Materials

Spent coffee grounds were collected from Guan Hin Coffee Powder Manufacturing (Pte.) Ltd. (Singapore). Dry polyvinyl alcohol (PVA) flakes were used to make the PVA solution. PVA flakes and methyltrimethoxysilane (MTMS) were purchased from Sigma Aldrich Pte. Ltd. (Singapore) and used as received. Both cotton pads and synthetic motor oil (Carlube 5w50) were purchased from a commercial market. All water used in the experimental work was deionized.

### 2.2. Fabrication of Hybrid Coffee-Cotton Aerogels

Spent coffee grounds were first weighed on a precision balance and then pounded with a suitable amount of water to make into paste. Cotton pads were cut into pieces of about 0.2 cm by 1.7 cm. Aqueous 5 wt.% PVA solution was prepared by dissolving PVA flakes into warm deionized (DI) water at 80 °C for 3 h stirring. Gel solution was prepared by mixing the spent coffee ground solution, PVA (5 wt.% concentration) and cotton fibers with fixed weight ratio of coffee powder. The resulting gel solution was sonicated for 30 min at 200W using a high surface sonication probe (UIP2000hdT, Hielscher-Ultrasound Technology, Teltow, Germany) to obtain a homogeneous gel solution free of bubbles. The solution was then cured to reactivate the PVA molecules to its dissolving temperature, allowing it to bond with the coffee particles and fibers in the solution. After curing, the solution was frozen. This freezing process caused the solution to gelate and get proper shapes. Freeze drying (with Toption, TPV-50F, Xi’an, China) the mould removed all the fluid presented through sublimation, with the chain network of the PVA, coffee powder and cotton fibers remaining. 

### 2.3. Surface Modification

The hydrophilic aerogel was placed in an air-tight container with open vials of MTMS and heated to 70 °C for 24 h in the oven (UN30, Memmert, Schwabach Germany). A hydrophobic aerogel was obtained after the deposition of MTMS vapor on the aerogel. 

### 2.4. Characterization 

The density and porosity of the aerogels were calculated using its weight and dimensions, density of spent coffee grounds, density of cotton fibers and density of PVA. The equation used to find the density of aerogels was:

(1) $$\mathrm{Density}\left(g/{\mathrm{cm}}^{3}\right)=\frac{\mathrm{Mass}}{\mathrm{Volume}}$$

The density of spent coffee ground, cotton fiber and PVA are 0.93 $g/{\mathrm{cm}}^{3}$ [20], 1.54 $g/{\mathrm{cm}}^{3}$ [21] and 1.91 $g/{\mathrm{cm}}^{3}$ [22], respectively. The porosity of aerogel was calculated using the following formula [14]:

(2) $$\mathrm{Porosity},\emptyset \;=\;100\;(1\;-\;\frac{\rho_{aerogel}}{\rho_{bulk}})$$

where $\rho_{aerogel}$ is the density of the aerogel and $\rho_{bulk}$ is the bulk density of the aerogel. The $\rho_{bulk}$ can be calculated from the following equation:

(3) $$\frac{C_{coffee\;powder}\;+\;C_{cotton\;fiber}\;+\;C_{PVA}}{\rho_{bulk}}\;=\;\frac{C_{coffee\;powder}}{\rho_{coffee\;powder}}+\frac{C_{cotton\;fiber}}{\rho_{cotton\;fiber}}+\frac{C_{PVA}}{\rho_{PVA}}$$

where $C_{coffee\;powder}$ is concentration of coffee powder (wt.%), $C_{cotton\;fiber}$ is concentration of cotton fiber (wt.%) and $C_{PVA}$ is concentration of PVA (wt.%).

Field emission scanning electron microscopy (FE-SEM S4300, Hitachi, Tokyo, Japan) was used to observe the surface morphologies of the aerogels. Water contact angle of the aerogels were determined using the VCA Optima goniometer (VCA Optima, AST Products Inc., Billerica, MA, USA). 

The thermal conductivity was conducted on the surface of the aerogels using the C-Therm TCi thermal conductivity analyzer (C-Therm Technologies, Fredericton, NB, Canada). Thermal stability of the aerogels was obtained with the TA Instruments Q500 Thermogravimetric Analyzer (TA Instruments, New Castle, DE, USA). 

The investigation of the oil absorption capability of the aerogels was conducted. The absorption capacity can be calculated as follows:

(4) $$Q=\frac{m_{i}-m_{f}}{m_{aerogel}}$$

where *Q* is absorption capacity, $m_{i}$ is the initial mass of the oil in the beaker, $m_{f}$ is the final mass of the oil in the beaker and $m_{aerogel}$ is the mass of the cut piece of aerogel.

The specific absorption was plotted against time to determine the absorption kinetics and were modelled using the first-pseudo order and second-pseudo order. 

The mechanical properties of the aerogels were also tested with an Instron 5500 Micrometer (Instron, Norwood, MA, USA). Compression test were carried out using cylindrical samples with diameter around 4 cm. The aerogels were compressed at a load rate of 1mm per minute using a 1000 N load cell. A stress against strain curve was plotted using the results obtained. 3–5 repeated samples was tested for each composition. 

## 3. Results and Discussion

### 3.1. Morphologies and Structures of Hybrid Aerogels.

**Figure 1:**
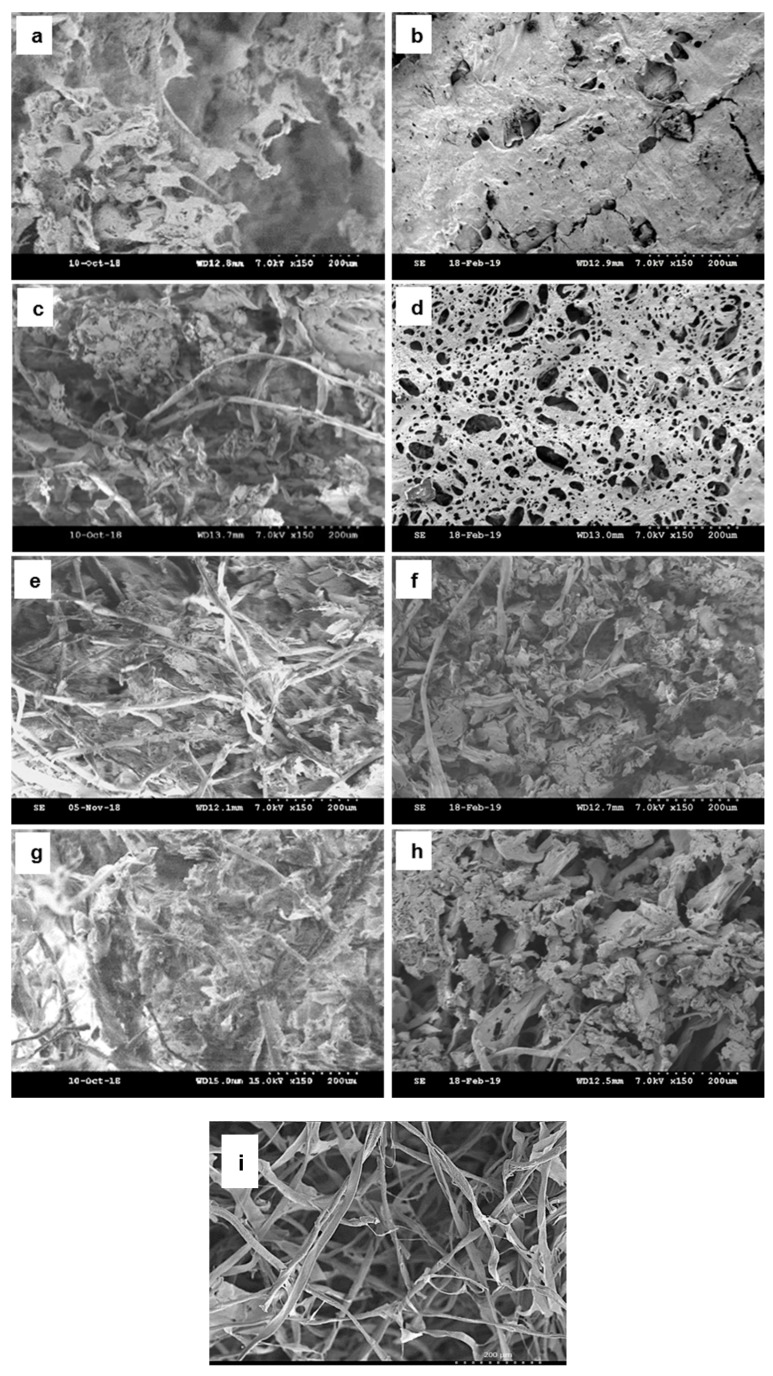
SEM images of coffee powder aerogel and increasing cotton fiber concentration hybrid cellulose-coffee aerogels: (**a**) top surface & (**b**) bottom surface of 2 wt.% coffee powder aerogel, (**c**) top surface & (**d**) bottom surface of 0.25 wt.% fiber + coffee powder aerogel, (**e**) top surface & (**f**) bottom surface of 0.50 wt.% fiber + coffee powder aerogel, (**g**) top surface, (**h**) bottom surface of 1.00 wt.% fiber + coffer powder aerogel & (**i**) cotton fiber aerogel.

The coffee aerogels formed between PVA and the coffee powder/cotton fibers are structured through hydrogen bonds. Figure 1 shows the SEM micrographs of the coffee powder aerogels with increasing concentration of cotton fibers (0, 0.25, 0.50 and 1.00 wt.%). The coffee concentration is kept at 2 wt.% and different structures on the top and bottom surfaces are observed. From SEM images, the coffee aerogels exhibit a highly porous network structure together with the distribution of PVA and cotton fibers connecting in between, indicating that the coffee powder/PVA successfully formed a three-dimensional porous network. For pure coffee aerogels without cotton fibers, only PVA is observed on the top surface of aerogel from SEM images (Figure 1a), while most of the coffee powder sink to the bottom of the aerogel and make the bottom surface rather smooth with tiny pores (Figure 1b). After adding in cotton fibers, the top surfaces of aerogels (Figure 1c,e,g) become more compacted with increasing concentration of fibers. On the other hand, the bottom surface (Figure 1d,f,h) of the samples show a compacted and rough bottom surface after cotton fibers’ reinforcement, which is similar to their top surface, implying that the fibers added help to hold and distribute the coffee powder more evenly in aerogels. 

In order to study the effect of cotton fiber concentration, the cotton fiber concentration is varied while fixing coffee ground concentration at 2 wt.% and PVA concentration at 1 wt.%. The Co represents the samples with cotton fiber concentration at 0 wt.%. 0.25Fib-Co, 0.5Fib-Co and 1.0Fib-Co represent samples with fiber concentrations at 0.25 wt.%, 0.50 wt.% and 1.00 wt.%, respectively. The coffee aerogels exhibit high porosity level (92.1–95.5%) and low densities (0.045 g/cm^3^), which are summarized in Table 1. It can be observed that with increasing the concentration of cotton fiber, while keeping the concentration of coffee and PVA constant, the aerogel density is decreased and the porosity is increased (i.e., density and porosity are inversely proportionate). Drawing from the observation in Figure 1, the density of the aerogels should increase as more cotton fiber are added, however, Table 1 shows the opposite trend (i.e., density decreased). The change of the density may cause by the shrinking of coffee aerogels, which can be observed from the change in volume (Table 1). This shrinking is due to the structure in pure coffee aerogel collapse, thus leading to a higher density and lower porosity. With increasing concentration of cotton, the shrinking of aerogel is reduced significantly. The addition of cotton fibers help to support and give structure to the aerogel, minimized the shrinkage, which explain the trend observed in Table 1. The lower in porosity observed in 1.0 Fib-Co compared to 0.5 Fib-Co is due to the presence of more fiber in the aerogel, thus occupying more space in the same volume, since the volume change remain relatively constant for both aerogels.

**Figure 2:**
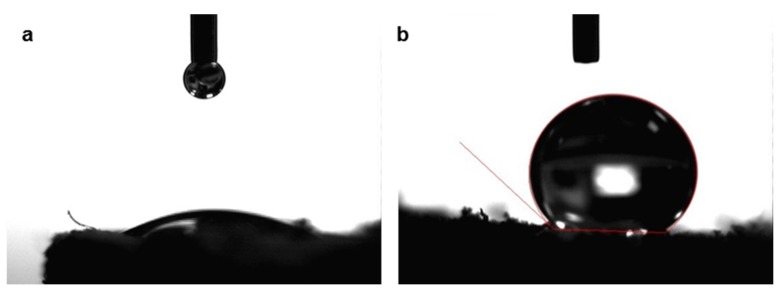
Hydrophobicity of silanized aerogels before (**a**) and after (**b**) MTMS coating.

The coffee powder aerogel and hybrid coffee-cotton aerogels are coated with MTMS via a gas deposition method. Figure 2 shows the water dots on the top of coffee aerogel before and after surface treatment. Hydrophobic coating is done successfully on the surface of coffee aerogels. The aerogel surface before the treatment is hydrophilic (Figure 2a), while a super-hydrophobic surface is observed after the treatment (Figure 2b). The effects of the different compositions on aerogel hydrophobicity are also investigated and shown in Table 2. All the coffee aerogels possess the water contact angle ranges from 125° to 139°. There is no significant difference between water contact angles of variation of coffee aerogel. In comparing to cotton aerogels and polymer aerogels, the coffee aerogels show the similar hydrophobic property with water contact angles after coating by MTMS [19,23].

### 3.2. Oil Absorption Capacities and Kinetics of the Coffee and Hybrid Aerogels

**Figure 3:**
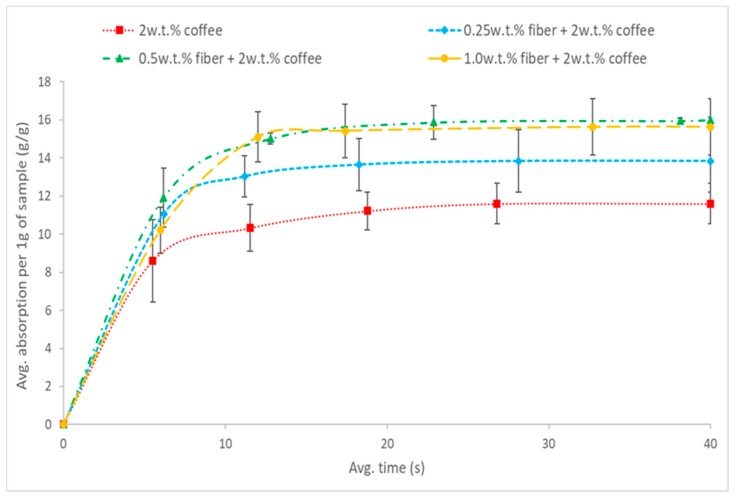
Oil absorption kinetics of 5w50 motor oil with coffee powder aerogel and increasing cotton fiber concentration hybrid cellulose-coffee aerogels.

**Figure 4:**
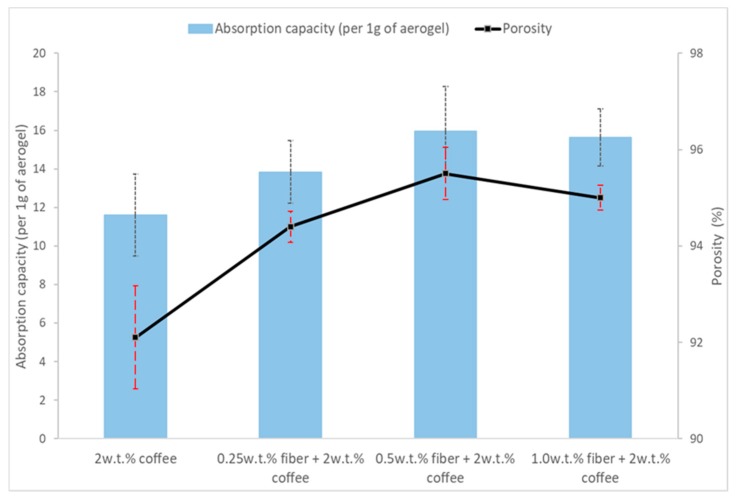
Oil absorption capacities of coffee powder aerogel and increasing cotton fiber concentration hybrid cellulose-coffee aerogels.

The oil absorption kinetics is carried out using the 5w50 motor oil, 3 samples has been tested for each composition. The oil absorption rates are observed to be very fast; equilibrium is reached after 15 s (Figure 3). The absorption kinetics of machine oil on different coffee-cotton aerogels are evaluated and summarized in Table 3. The maximum absorption capacity of the coffee aerogel increases with increasing cotton fibers concentration, and keep similar after it reaches 0.50 and 1.00 wt.% (Figure 4). The cotton fibers help to improve the coffee powder uniformity and increase the porosity of aerogels, thus there is more space for absorbing oil and this leads to a higher oil absorption capacity of the hybrid aerogels. On the other hand, after increasing cotton fiber concentration from 0.50 wt.% to 1.00 wt.%, the density of aerogel has decreased, and this may induce the slightly lower oil capacity.

To determine the absorption model used for oil, the pseudo first order and pseudo second order models were used. For pseudo first order model, the following equation was used:

(5) $$\ln\frac{Q_{m}}{Q_{m}-Q_{t}}\;=\;k_{1}t$$

where $Q_{m}$ is the maximum absorption capacity, $Q_{t}$ is the oil absorption at time, *t*, and $k_{1}$ is the constant rate of absorption. $k_{1}$ is the gradient which can be obtained by plotting $\ln\frac{Q_{m}}{Q_{m}-Q_{t}}$ against time, *t*.

For the pseudo second order model, the following equation was used:

(6) $$\frac{t}{Q_{t}}\;=\;\frac{1}{Q_{m}}t\;+\;\frac{1}{k_{2}Q_{m}^{2}}$$

Plotting $\frac{t}{Q_{t}}$ against time, $k_{2}$ can be determined by rearranging $\frac{1}{k_{2}Q_{m}^{2}}$ which is the y-intercept of the graph.

Fitting of the experimental absorption kinetics data to the pseudo-first and pseudo-second order models is compared in Table 3. It is noted that the correlation coefficient R^2^ in the pseudo-second order model is greater than that in the pseudo-first order model, as R^2^ = 0.9993 and 0.8885, respectively. Therefore, it is deemed that the pseudo-second order model is a better model in predicting accurately the oil absorption behavior of the coffee-cotton hybrid aerogels. The fitting experiment absorption kinetics data on the coffee aerogels at different cotton fiber concentration also prove that the pseudo-second order model can predict better oil absorption behavior in this work.

### 3.3. Thermal Properties of the Hybrid Coffee-Cotton Aerogels

**Figure 5:**
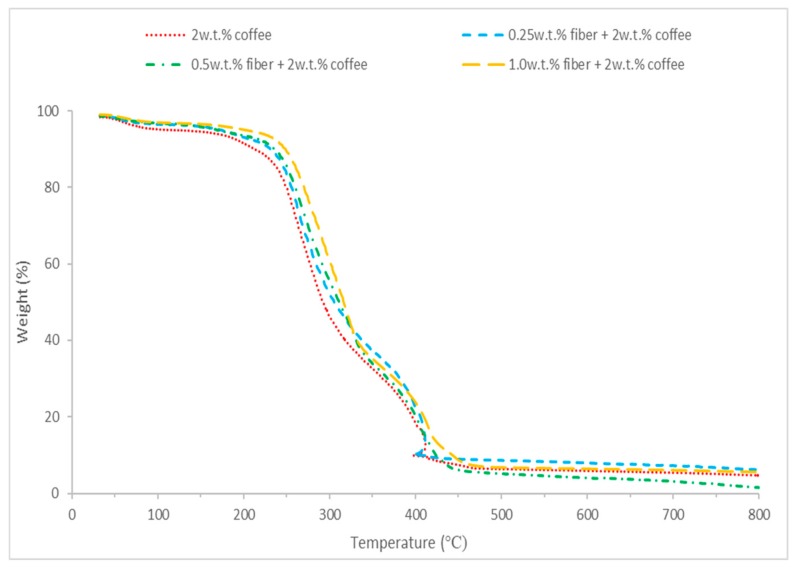
TGA curve of coffee powder aerogel and increasing cotton fiber concentration hybrid cellulose-coffee aerogels.

The thermal stability of the coffee aerogels was investigated by TGA. As shown in Figure 5, the first stage is the loss of moisture, which was observed to occur up to 100 °C for all aerogels. In the second stage, the aerogels will start to degrade thermally at around 200 °C to about 430 °C due to decomposition of coffee structure in SCG like hemicellulose, lignin and cotton cellulose compound like levoglucosan [24,25]. The weight loss occurs at about 300 °C stands for decomposition of PVA on the surface of the coffee aerogels. It is observed that the aerogels do not completely decomposed at the last stage ranging from 430 °C to 800 °C. This is due to char consolidation because of slow decomposition of residual solid [26] which is a result of lignin components [27]. Therefore, due to the slow carbonization of lignin, carbon could be the main component of the char production [28,29]. 

The thermal conductivities, K of the various coffee aerogel with different cotton fiber concentrations is determined and summarized in Table 4. At the ambient temperature and pressure conditions, the highly porous coffee aerogels possess low density, which contribute to its low thermal conductivity. It is observed that the top surface of the aerogels shows an increasing thermal conductivity and the bottom surface of the aerogels shows the opposite with increasing cotton fiber concentration. Differences between top and bottom surface are decreased until both thermal conductivity values are similar. This is because the uniformity of coffee powder has improved significantly as higher concentration of fibers are added in. This has been further supported by the SEM images in Figure 1. Although there are differences between thermal conductivities for the top and bottom surfaces of the aerogels, the overall average thermal conductivity remains relatively constant (0.045 W/mK) for the hybrid-cellulose coffee aerogels. 

### 3.4. Mechanical Properties of the Hybrid Coffee-Cotton Aerogels

**Figure 6:**
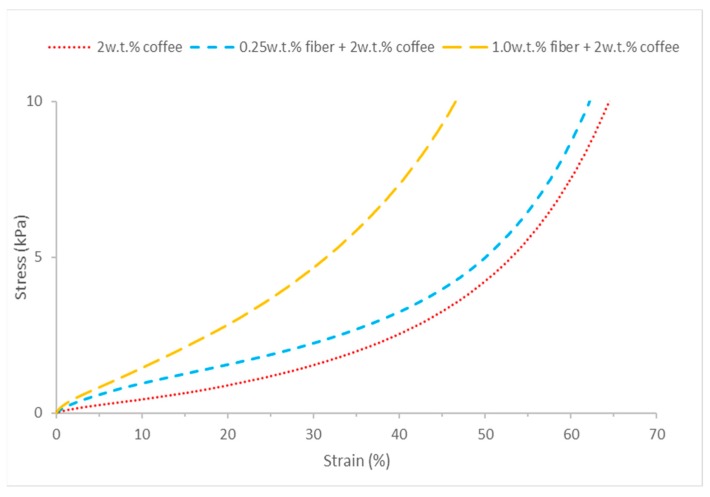
Stress-strain curve of coffee powder aerogel and increasing cotton fiber concentration hybrid cellulose-coffee aerogels.

Compressive tests are conducted to understand the mechanical properties of coffee-cotton hybrid aerogels, with different cotton fiber content. The compressive Young’s modulus indicates the stiffness of solid materials and the value depends on the structure of the materials. Aerogels with more compactness will give a higher compressive Young’s modulus. The compressive stress-strain curves and compressive Young’s modulus, E of the hybrid aerogels are shown in Figure 6 and Table 5. The results indicate coffee aerogels generally exhibiting low elasticity in the range of 5.4–15.6 kPa. The elastic modulus of the coffee aerogel increases with an increase in its density.

The compression strength and modulus increase significantly with cotton fiber, and 1.0 Fib-Co gives the highest value in both. This may due to the entanglement of cotton fibers within aerogel, which help to improve strength to overall structure. However, the modulus of 0.5 Fib-Co is slightly lower compared to the other hybrid aerogels due to its lowest density and less packed structure. 

## 4. Conclusions

Cost effective and fully biodegradable hybrid coffee-cotton aerogels were successfully fabricated using environmental friendly raw materials and processes. The uniformity of coffee powder has been improved significantly after adding in cotton fibers. A cotton fiber concentration at 0.50 wt.% achieved the lowest density, which might be due to the fact the uniformity of the samples is the best. The hybrid aerogels are investigated at different cotton concentrations and show excellent properties, including oil absorption capacities, thermal insulation, and mechanical properties. The superhydrophobic hybrid aerogel with 2 wt.% of coffee and 0.50 wt.% of cotton fibers possesses the highest porosity (95.5%) and oil absorption capacity (16 g/g). The thermal conductivity of the hybrid aerogel is in the range 0.037–0.045 W/mK and the compressive Young’s modulus reaches a highest value of 15.6 kPa. The developed hybrid aerogel is a promising environmentally-friendly material for potential applications in thermal insulation, oil absorption and lightweight composites.

## Figures and Tables

**Table 1:** Material composition of ground coffee powder and hybrid cellulose-coffee aerogels.

Samples	Spent Coffee Ground (wt.%)	Cotton Fiber (wt.%)	PVA (wt.%)	Density (mg/cm^3^)	Porosity (%)	Volume Change (%)
Co	2.0	0	1.0	79.7 ± 10.7	92.1 ± 1.1	62.5 ± 5.0
0.25 Fib-Co	2.0	0.25	1.0	57.3 ± 3.3	94.4 ± 0.3	43.6 ± 2.4
0.5 Fib-Co	2.0	0.50	1.0	45.8 ± 6.1	95.5 ± 0.5	28.5 ± 6.5
1.0 Fib-Co	2.0	1.00	1.0	55.3 ± 2.8	95.0 ± 0.3	28.5 ± 3.8

**Table 2:** Angles of water droplets on hydrophobic silanized coffee powder aerogel and increasing cotton fiber concentration hybrid cellulose-coffee aerogels.

	Co	0.25 Fib-Co	0.5 Fib-Co	1.0 Fib-Co
Angle (°)	136.8 ± 3.7	124.7 ± 2.5	139.1 ± 4.6	132.2 ± 3.7

**Table 3:** Summary of maximum oil absorption capacities and absorption rate constants for coffee powder aerogel and increasing concentration of cotton fiber hybrid cellulose-coffee aerogels.

		Co	0.25 Fib-Co	0.5 Fib-Co	1.0 Fib-Co
Maximum absorption capacity	$Q_{m}$ (g/g)	11.6	13.8	16.0	15.6
Pseudo first order	$R^{2}$	0.8885	0.9595	0.9476	0.9912
	$k_{1}$	0.2954	0.3171	0.1564	0.2501
Pseudo second order	$R^{2}$	0.9993	0.9993	0.9990	0.9948
	$k_{2}$	0.0313	0.0396	0.0289	0.0217

**Table 4:** Thermal conductivities of coffee powder aerogel and increasing cotton fiber concentration hybrid cellulose-coffee aerogels measured at ambient temperature (25 °C).

Thermal Conductivity, K_avg_ (W/mK)	Co	0.25 Fib-Co	0.5 Fib-Co	1.0 Fib-Co
Top surface	0.037 ± 0.001	0.038 ± 0.001	0.041 ± 0.001	0.042 ± 0.001
Bottom surface	0.062 ± 0.001	0.047 ± 0.002	0.045 ± 0.001	0.044 ± 0.002
Avg. of both surfaces	0.050 ± 0.001	0.043 ± 0.001	0.043 ± 0.001	0.043 ± 0.003

**Table 5:** The compression Young’s modulus of coffee-cotton aerogel.

Samples	Spent Coffee Ground (wt.%)	Cotton Fiber (wt.%)	Density (mg/cm^3^)	Compressive Young’s Modulus E (kPa)
Co	2	0	79.9 ± 10.7	5.41 ± 0.09
0.25 Fib-Co	2	0.25	57.3 ± 3.3	7.69 ± 0.01
0.5 Fib-Co	2	0.50	45.8 ± 6.1	6.34 ± 0.06
1 Fib-Co	2	1.00	55.3 ± 2.8	15.62 ± 0.06
